# Molecular genetic characteristics of thymic epithelial tumors with distinct histological subtypes

**DOI:** 10.1002/cam4.5795

**Published:** 2023-03-14

**Authors:** Jun Yang, Biao Zhang, Wenyan Guan, Zhiwen Fan, Xiaohong Pu, Linyue Zhao, Wen Jiang, Weijing Cai, Xueping Quan, Shuying Miao, Ling Nie, Lu He

**Affiliations:** ^1^ Department of Pathology, Nanjing Drum Tower Hospital The Affiliated Hospital of Nanjing University Medical School Nanjing China; ^2^ Shanghai Tongshu Biotechnology Co., Ltd. Shanghai China

**Keywords:** amplified segments, copy number variation burden, thymic carcinomas, thymic epithelial tumors, thymomas, weighted genome instability index

## Abstract

**Background:**

Due to the low incidence and histological heterogeneity, the molecular features and underlying carcinogenic mechanisms of thymic epithelial tumors (TETs) are yet to be fully elucidated, especially for different subtypes of TETs.

**Methods:**

Tumor tissue samples of 43 TETs with distinct histological subtypes were collected. We analyzed the molecular characteristics in different subtypes based on whole exome sequencing data.

**Results:**

The mutational profiles of the different subtypes of TETs varied. Compared with thymomas, thymic carcinomas (TCs) had a higher mutation frequency of *MYO16* (33% vs. 3%, *p* = 0.024) and a lower frequency of *ZNF729* mutations (0% vs. 35%, *p* = 0.044). No significant difference was observed in the median tumor mutation burden across different subtypes. The value of copy number variation burden, weighted genome instability index, and the number of amplified segments were all higher in TCs than thymomas, and they also tended to be higher in B3 thymoma than in non‐B3 thymomas, while they had no significant differences between B3 thymoma and TCs. Clustering analyses revealed that Wnt, MAPK, Hedgehog, AMPK, and cell junction assembly signaling pathways were exclusively enriched in non‐B3 thymomas, lysine degradation pathway in B3 thymoma, and extracellular matrix‐receptor (ECM‐receptor) interaction, positive regulation of cell cycle process, and activation of innate immune response pathways in TCs.

**Conclusions:**

This study revealed distinct molecular landscapes of different subtypes of TETs, suggesting diverse pathogenesis of non‐B3 thymomas, B3 thymomas, and TCs. Our findings warrant further validation in future large‐scale studies and may provide a theoretical basis for potential personalized therapeutic strategies.

## Graphical abstract

The value of copy number variation (CNV) burden, weighted genome instability index (wGII), and the number of amplified segments were all higher in TCs than thymomas, and they also tended to be higher in B3 thymoma than in non‐B3 thymomas.

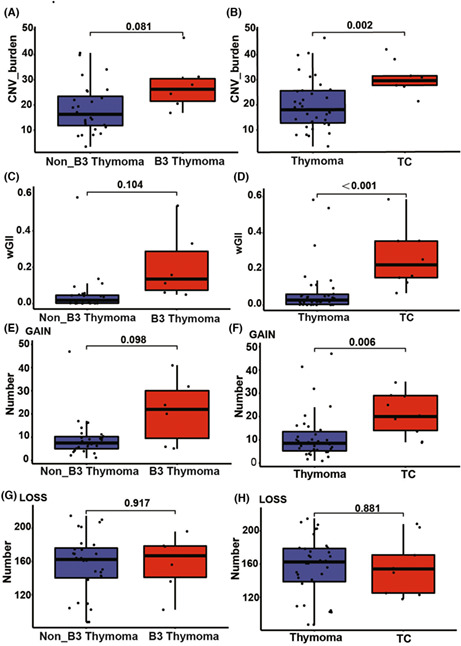

## 1 INTRODUCTION

Thymic epithelial tumors (TETs) comprise a morphologically and clinically heterogeneous group of rare neoplasms originating from the thymus, including thymomas, thymic carcinomas (TCs), and thymic neuroendocrine tumors.^1^ The estimated incidence of TETs is 0.15 per 100,000 person‐years in the United States,^2^ and 0.393 per 100,000 person‐years in China.^3^ According to the World Health Organization (WHO) classification, TETs are classified into thymomas (types A, AB, B1, B2, and B3) and thymic carcinoma by the morphology of epithelial cells and the ratio of lymphocytes to epithelial cells.^1^, ^4^ Different histologic subtypes have been reported to bear distinct prognoses.^5^, ^6^ For example, the 5‐year survival rate of thymomas was 69%, which was better than that of TCs (36%).^6^

Previous studies have also found that different subtypes of TETs exhibit distinct molecular profiles.^5^, ^7^, ^8^, ^9^ For example, there is a high frequency of recurrent mutations in the GTF2I gene in type A and AB thymomas.^7^, ^8^ *HRAS* Mutations are also largely restricted to type A and AB thymomas, while *NRAS* and *TP53* mutations are much more common in type B2 and B3 thymomas and TCs.^7^ However, data regarding the integrated genomic landscape of these different subtypes of TETs remain limited and small scale due to the relatively low incidence. The etiology of TETs needs to be further elucidated.

The modalities to treat TETs mainly included surgery, radiotherapy, and chemotherapy. Surgery is the footstone in the cure of early‐stage TETs, while platinum‐based chemotherapy is considered the standard of care for advanced or metastatic TETs.^10^ Nevertheless, there is a paucity of standard salvage treatments after the failure of platinum‐based chemotherapy.^10^, ^11^ Although lenvatinib and everolimus may be promising in treating TETs and pembrolizumab have been recommended by National Comprehensive Cancer Network (NCCN) guidelines as second‐line systemic therapy for refractory TCs, the data concerning therapeutic efficacies are warranted to be validated in larger cohorts.^12^, ^13^, ^14^

In this context, a deeper understanding of the molecular characteristics of each subtype in TETs might facilitate unraveling specific pathogenesis, guiding potential personalized therapy, and exploring novel biomarkers to predict prognosis and response to treatment. Therefore, we aim to investigate and compare the mutation profiles, immunotherapeutic response‐related indicators, and enriched signaling pathways in various subtypes of TETs by whole exome sequencing (WES) in this study.

## 2 MATERIALS AND METHODS

### 2.1 Patients and sample collection

A total of 43 patients with TETs diagnosed between February 2018 and December 2020 at Nanjing Drum Tower Hospital were enrolled in the study, including 34 thymomas and nine TCs. The histologic subtypes of these cases were classified following the 2021 World Health Organization (WHO) classification of the thymus. None of them underwent neoadjuvant treatment before surgery. The formalin‐fixed and paraffin‐embedded (FFPE) tumor tissue sample for each patient was obtained by surgical excision before any further adjuvant therapies and tested by WES (Shanghai Tongshu Biotechnology Co., Ltd.). The normal control samples were not available for the cases included in this study. The clinicopathological information was retrospectively reviewed including age, gender, and histological classification.

### 2.2 Whole exome sequencing

We extracted DNA from FFPE tissue samples using the Tissue Kit (69504, QIAGEN) following the manufacturer's protocols. Tissue sections were examined by two pathologists independently. Samples with at least 30% tumor cells were selected, 93% of which contained ≥50% tumor cells, and 86% contained ≥80% tumor cells. DNA was isolated by targeted capture pull‐down and exon‐wide libraries were generated from native DNA using the xGen® Exome Research Panel (Integrated DNA Technologies) and TruePrep DNA Library Prep Kit V2 for Illumina (#TD501, Vazyme). Paired‐end sequencing was performed by NovaSeq 6000. The sequencing data were then aligned to the human reference genome (NCBI build 37) using the Burrows–Wheeler Aligner (BWA), and polymerase chain reaction duplicates were sorted and removed by sambamba.

### 2.3 Data processing

Somatic single nucleotide variants (SNVs), insertions, and deletions (INDELs) were detected using Mutect2. A panel of normal was created by in‐house healthy individuals to filter germline variants using the tumor‐only mode of GATK Mutect2 (4.1.8.1). Variants and polymorphisms were annotated using Annovar. INDEL calling was limited to non‐homopolymer and non‐repeat regions. A minimum of two reads supporting the variant allele and ≥10× mapped depth are required for somatic INDEL covering ≥9 bases calling. A minimum of four reads supporting the variant allele, and ≥10× mapped depth are required for somatic INDEL covering <9 bases calling. A minimum of three reads supporting the variant allele and ≥10× mapped depth are required for somatic SNV calling. Variants with MAF >1% in the ExAC, gnomAD, esp6500 databases, and ChinaMap were filtered out as common benign/likely benign germline variants. The limit of detection of variants was set to be variant allele frequency (VAF) being 1%. SNVs and INDELs absent in the COSMIC database were also filtered out. Somatic copy number variations (CNVs) were analyzed using FACETS, and the identified CNVs were used in further analysis. Weighted genome instability index (wGII) was analyzed as described in Burrell et al.'s work.^15^

### 2.4 Gene enrichment analysis

The Kyoto Encyclopedia of Genes and Genomes (KEGG)/gene ontology (GO) enrichment analysis was performed by the Cluster Profiler R package. The list of gene IDs was used as the input file. The Benjamini–Hochberg method calculated adjusted *p* ‐values, and statistical significance was declared at an adjusted *p‐*value <0.05. The results of enrichment analyses were visualized with the ggplot2 R package.

### 2.5 Statistical analysis

Fisher's exact test was applied to compare the categorical variables between the groups. All *p*‐values represented were two‐sided, and *p* < 0.05 was considered statistically significant.

## 3 RESULTS

### 3.1 Demographic and clinicopathological information

Of 43 patients with TETs, the median age was 62 (range, 26–81) years, and 23 (53.19%) were males. The majority of these patients were diagnosed with thymoma, among which seven patients were with type A, eight with type AB, four with type B1, seven with type B2, six with type B3, and two with combined type B. The remaining nine patients were with TCs. As to comorbidities, three cases with type B3 were diagnosed with Myasthenia Gravis, and six cases with non‐B3 TETs complained of mild fatigue. Other cases in this study did not report any other autoimmune diseases. Detailed clinicopathological characteristics were summarized in Table 1.

**TABLE 1:** Clinicopathological characteristics of 43 cases with thymic epithelial tumors.

Characteristics	*n* (%)
Total number	43
Age (years), median (range)	62 (26–81)
Gender	
Male	23 (53.5)
Female	20 (46.5)
Histological subgroup	
Thymoma (total 34)	
Type A	7 (16.3)
Type AB	8 (18.6)
Type B1	4 (9.3)
Type B2	7 (16.3)
Type B3	6 (14.0)
Combined B	2 (4.7)
Thymic carcinoma (total 9)	9 (20.9)

### 3.2 Mutation landscape of TETs with distinct subtypes

**FIGURE 1:**
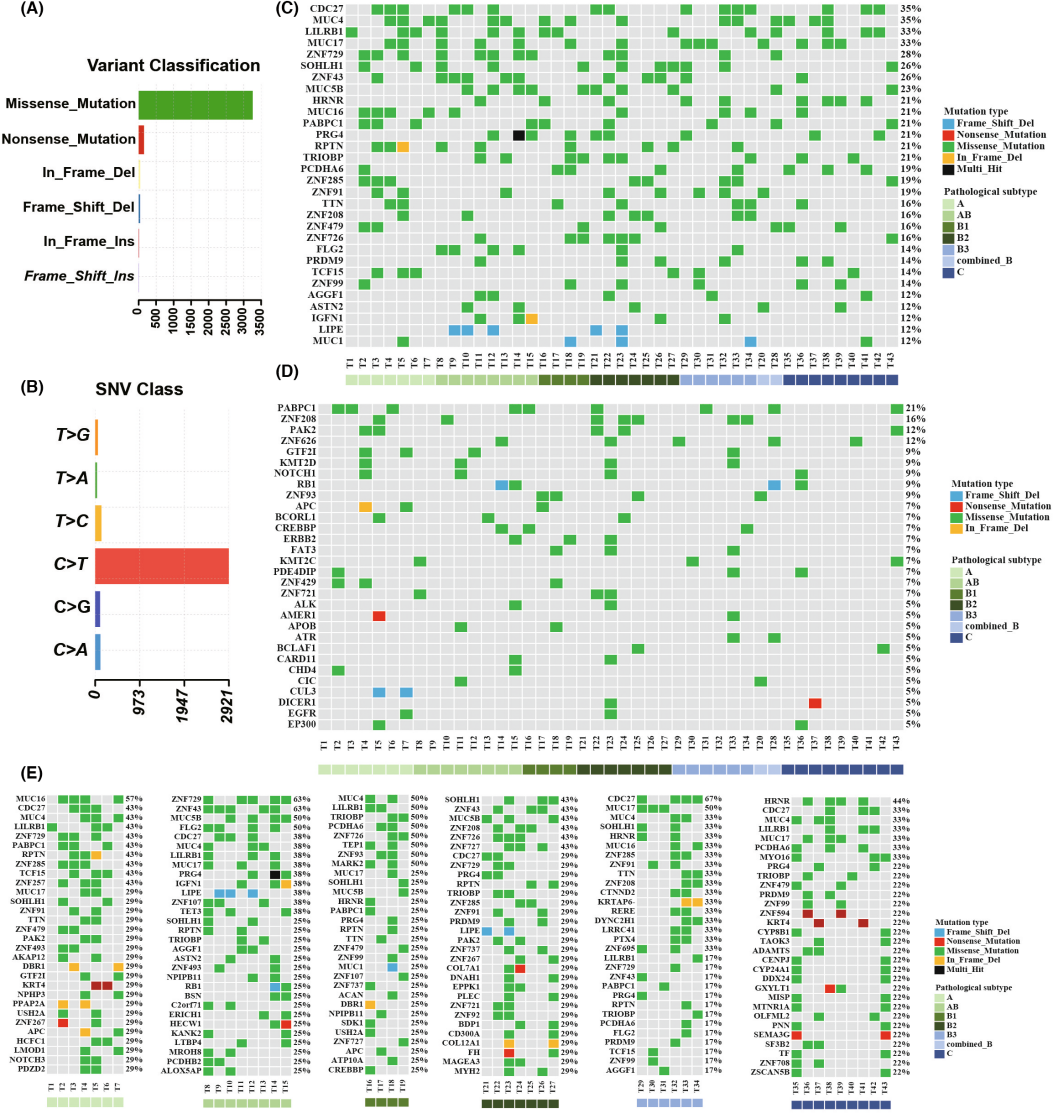
The mutation landscapes of 43 cases with thymic epithelial tumors (TETs). (A) The distribution of variant types in the whole cohort. (B) The distribution of single nucleotide variants classes in the whole cohort. (C) The top 30 frequently mutated genes of the whole cohort. (D) The top 30 frequently mutated driver genes of the whole cohort. (E) The mutation landscapes of each subtype of TETs.

**FIGURE 2:**
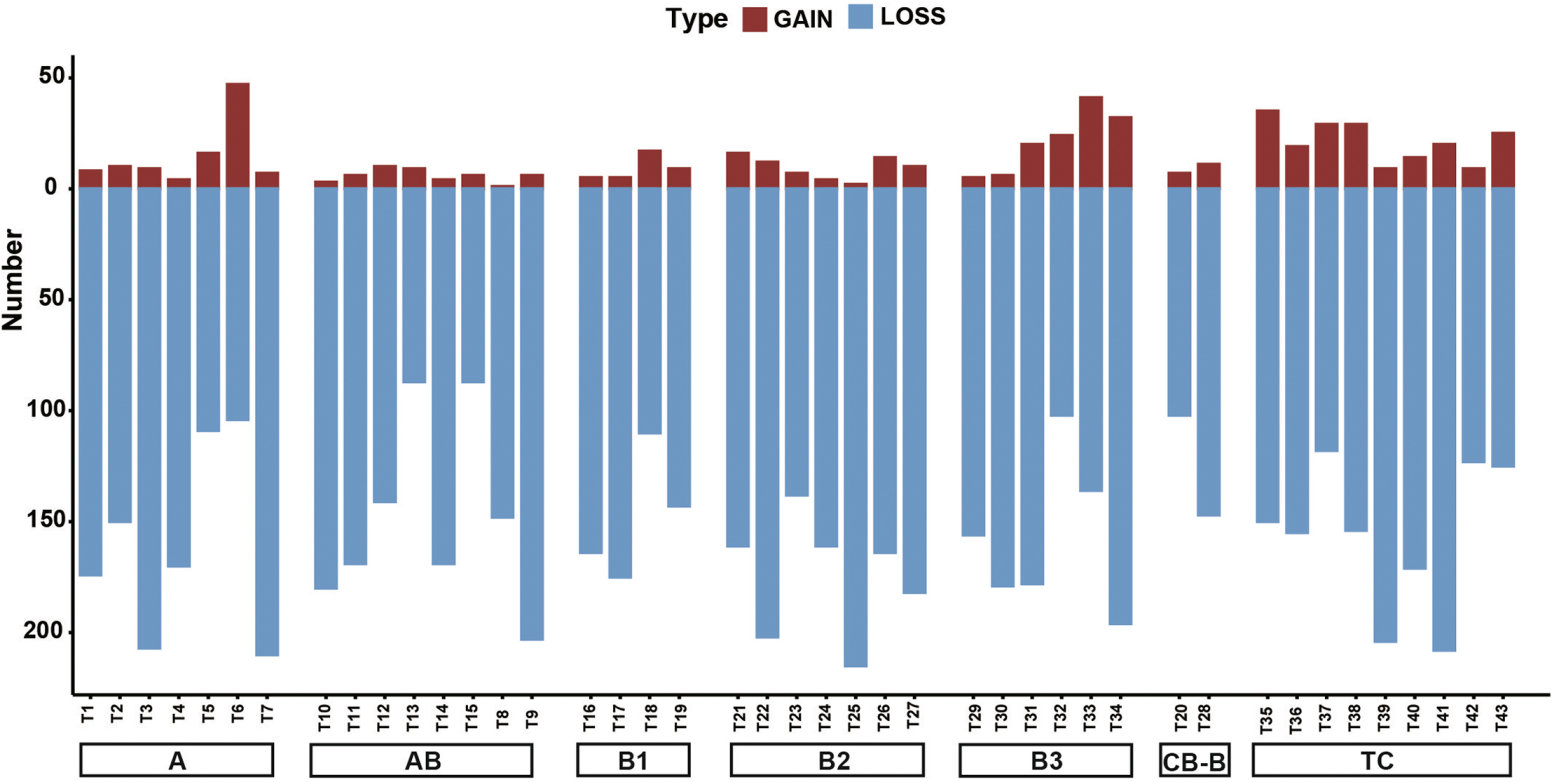
The number of amplified and deleted segments for each case with thymic epithelial tumors. Gain, amplified segments; Loss, deleted segments. CB‐B, combined type B.

By performing WES, missense mutations were predominantly detected in these patients with TETs (Figure 1A), and most of them were C > T substitutions (Figure 1B). For the whole cohort, the top three recurrently mutated genes included *CDC27* (35%), *MUC4* (35%), and *LILRB1* (33%) (Figure 1C). The frequently mutated driver genes included *PABPC1* (21%), *ZNF208* (16%), and *PAK2* (12%) (Figure 1D). For the different subtypes, mutational profiles varied as shown in Figure 1E. The most commonly mutated genes comprised *MUC16*, *CDC27*, and *MUC4* in the patients with type A thymoma, *ZNF729*, *ZNF43*, and *MUC5B* in type AB thymoma, *MUC4*, *LILRB1*, and *TRIOBP* in type B1 thymoma, *SOHLH1*, *ZNF43*, and *MUC5B* in type B2 thymoma, *CDC27*, *MUC17*, and *MUC4* in type B3 thymoma, *HRNR*, *CDC27*, and *MUC4* in TCs. Besides, all the cases had a larger number of deleted segments than amplified segments regardless of the histologic subtypes (Figure 2 **)**.

### 3.3 Comparison of genetic characteristics in TETs with distinct subtypes

**FIGURE 3:**
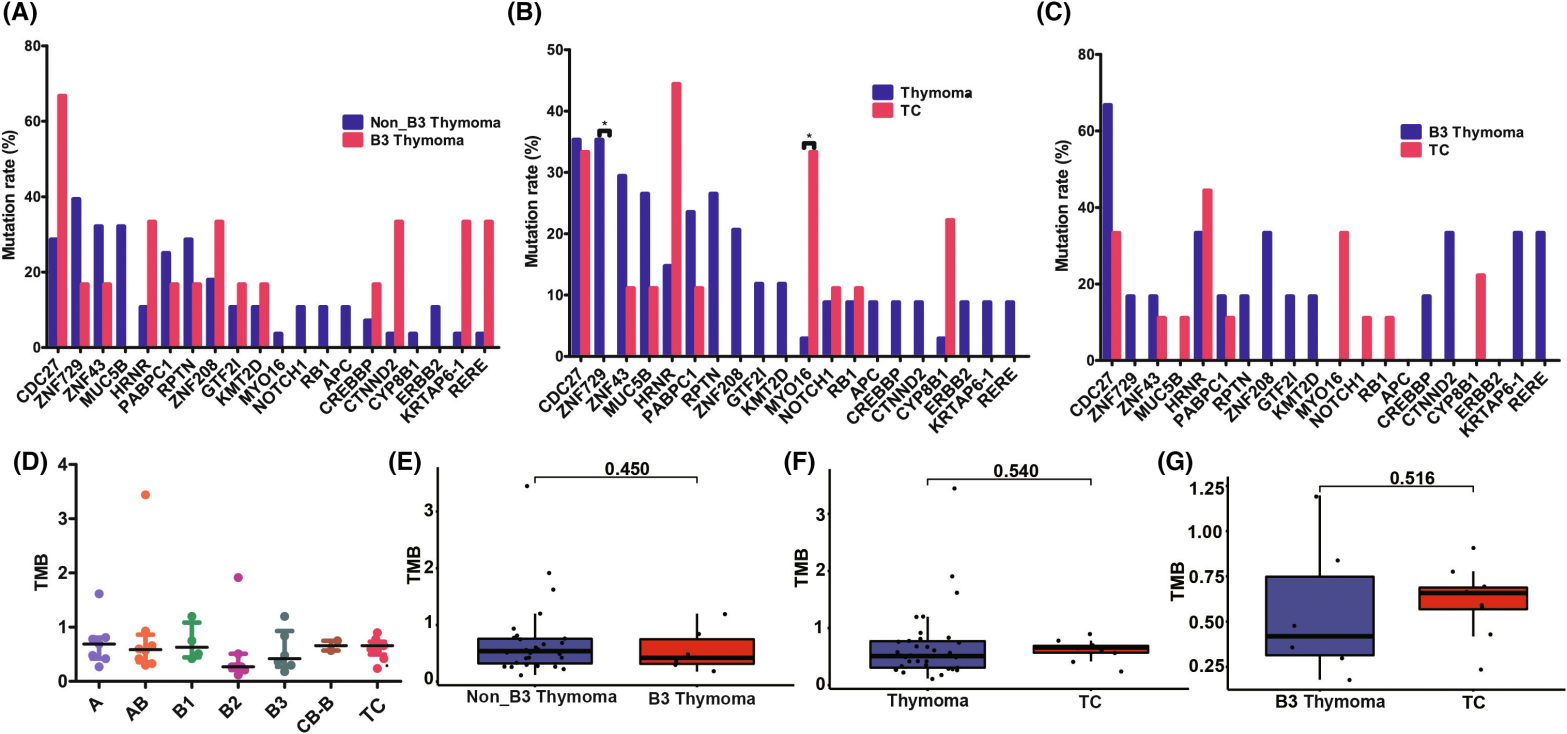
Comparisons of gene mutational frequencies and tumor mutation burden (TMB) among thymic epithelial tumors (TETs) with distinct subtypes. (A) Comparison of gene mutational frequencies between B3 thymoma and non‐B3 thymomas. (B) Comparison of gene mutational frequencies between thymomas and thymic carcinoma (TCs). (C) Comparison of gene mutational frequency between B3 thymoma and TCs. (D) TMB in type A, AB, B1, B2, B3, combined‐B TETs, and TCs. (E) Comparison of TMB between B3 thymoma and non‐B3 thymomas. (F) Comparison of TMB between thymomas and TCs. (G) Comparison of TMB between B3 thymoma and TCs (*p < 0.05).

The genetic characteristics were then compared among different subtypes. As shown in Figure 3A, the mutation frequencies of *CDC27* (67% vs. 29%), *CTNND2* (33% vs. 4%), *KRTAP6‐1* (33% vs. 4%), and *RERE* (33% vs. 4%) trended to be higher in type B3 thymoma than other subtypes of thymomas, while the mutation frequency of *MUC5B* (0% vs. 32%) trended to be lower in type B3 thymoma. The insignificant difference was probably due to the limited number of patients. Additionally, patients with TCs had a higher frequency of *MYO16* mutation (33% vs. 3%, *p* = 0.024) and a lower frequency of *ZNF729* mutation (0% vs. 35%, *p* = 0.044) than those with thymomas (Figure 3B). Similarly, the mutation frequency of *MYO16* had a trend to be higher in TCs than in B3 thymomas (33% vs. 0%, Figure 3C).

The tumor mutation burden (TMB) values of different subtypes were presented in Figure 3D and Table 2. No significant difference regarding TMB was observed between type B3 thymoma and non‐B3 thymomas (0.42 vs. 0.54, *p* = 0.450), TCs and thymomas (0.66 vs. 0.51, *p* = 0.540), B3 thymoma and TCs (0.42 vs. 0.66, *p* = 0.516), respectively (Figure 3E–G).

**TABLE 2:** The TMB‐ and CNV‐related indicators among distinct subtypes of TETs.

	TMB	CNV burden	wGII	Gain	Loss
A (range)	0.69 (0.27–1.62)	13.29 (8.58–40.61)	0.04 (0.001–0.58)	9.00 (4.00–47.00)	170.00 (104.00–210.00)
AB (range)	0.58 (0.30–3.44)	13.27 (3.42–39.81)	0.02 (0.003–0.05)	6.00 (1.00–10.00)	158.5 (87.00–203.00)
B1 (range)	0.63 (0.42–1.20)	15.02 (7.98–25.71)	0.04 (0.002–0.05)	7.00 (5.00–17.00)	153.5 (110.00–175.00)
B2 (range)	0.27 (0.12–1.92)	22.77 (16.22–34.04)	0.02 (0.002–0.14)	10.0 (2.00–16.00)	164.0 (138.00–215.00)
B3 (range)	0.42 (0.18–1.20)	26.22 (16.85–46.66)	0.14 (0.05–0.54)	22.00 (5.00–41.00)	167.0 (102.00–196.00)
TCs (range)	0.66 (0.24–0.90)	29.51 (21.31–42.00)	0.22 (0.07–0.59)	20.00 (9.00–35.00)	154.00 (118.00–208.00)
non‐B3 (range)	0.54 (0.12–3.44)	16.28 (3.42–40.61)	0.02 (0.001–0.58)	7.50 (1.00–47.00)	162.50 (87.00–215.00)
Thymomas (range)	0.51 (0.12–3.44)	18.05 (3.42–46.66)	0.03 (0.001–0.58)	8.50 (1.00–47.00)	162.50 (87.00–215.00)
non‐B3 vs. B3 *p* value	0.450	0.081	0.104	0.098	0.917
TC vs. thymoma *p* value	0.540	0.002	<0.001	0.006	0.881
B3 vs. TCs *p* value	0.516	0.388	0.328	0.961	0.776

Abbreviations: CNV, copy number variations; Gain, amplified segments; Loss, deleted segments; TC, thymic carcinoma; TMB, tumor mutation burden.

**FIGURE 4:**
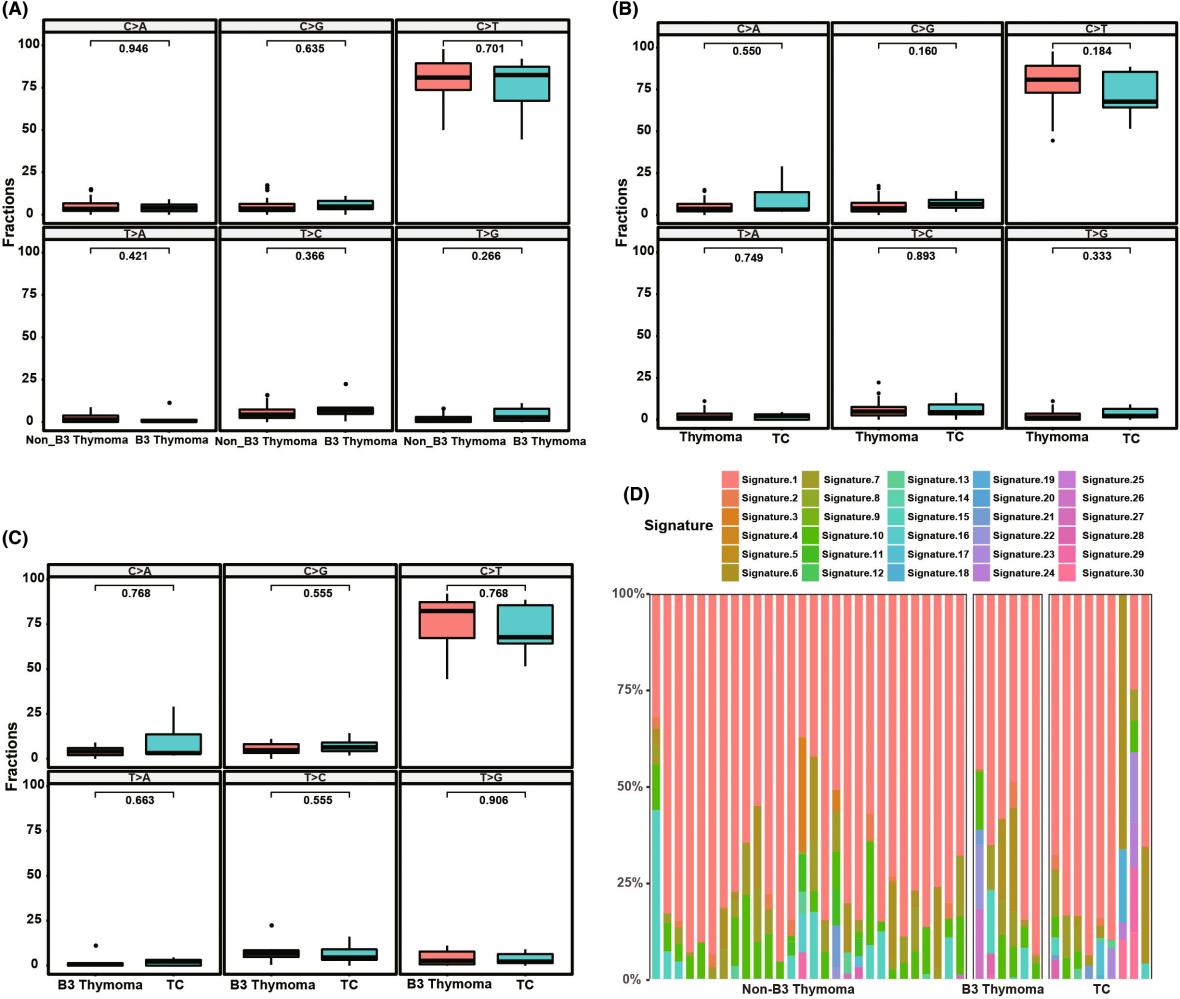
Relative contributions of six substitution subtypes for single nucleotide variants (SNV) and mutational signatures in thymic epithelial tumors with distinct subtypes. (A) Relative contributions of six substitution subtypes for SNV in B3 thymoma and non‐B3 thymomas. (B) Relative contributions of six substitution subtypes for SNV in thymomas and thymic carcinoma (TCs). (C) Relative contributions of six substitution subtypes for SNV in B3 thymoma and TCs. (D) Relative contributions of mutational signatures in each group.

The relative distributions of six substitution subtypes for SNV and mutational signatures across the distinct subtypes of TETs indicated that C > T substitutions were predominantly detected in thymomas and TCs **(**Figure 4A–C **)**. The percentage of C > T substitutions seemed to decrease in TCs more than that in B3 thymomas and non‐B3 thymomas (average: 73% vs. 75% and 80%) with no significant difference. Additionally, the mutational signature analysis identified signature 1, signature 6, signature 7, signature 10, and signature 15 across the whole spectrum of thymomas and TCs (Figure 4D). The percentages of signature 6, which is associated with defective DNA mismatch repair, trended to be higher in TCs and B3 thymomas than that in non‐B3 thymomas (average: 12% and 8% vs. 4%) with no significant difference.

**FIGURE 5:**
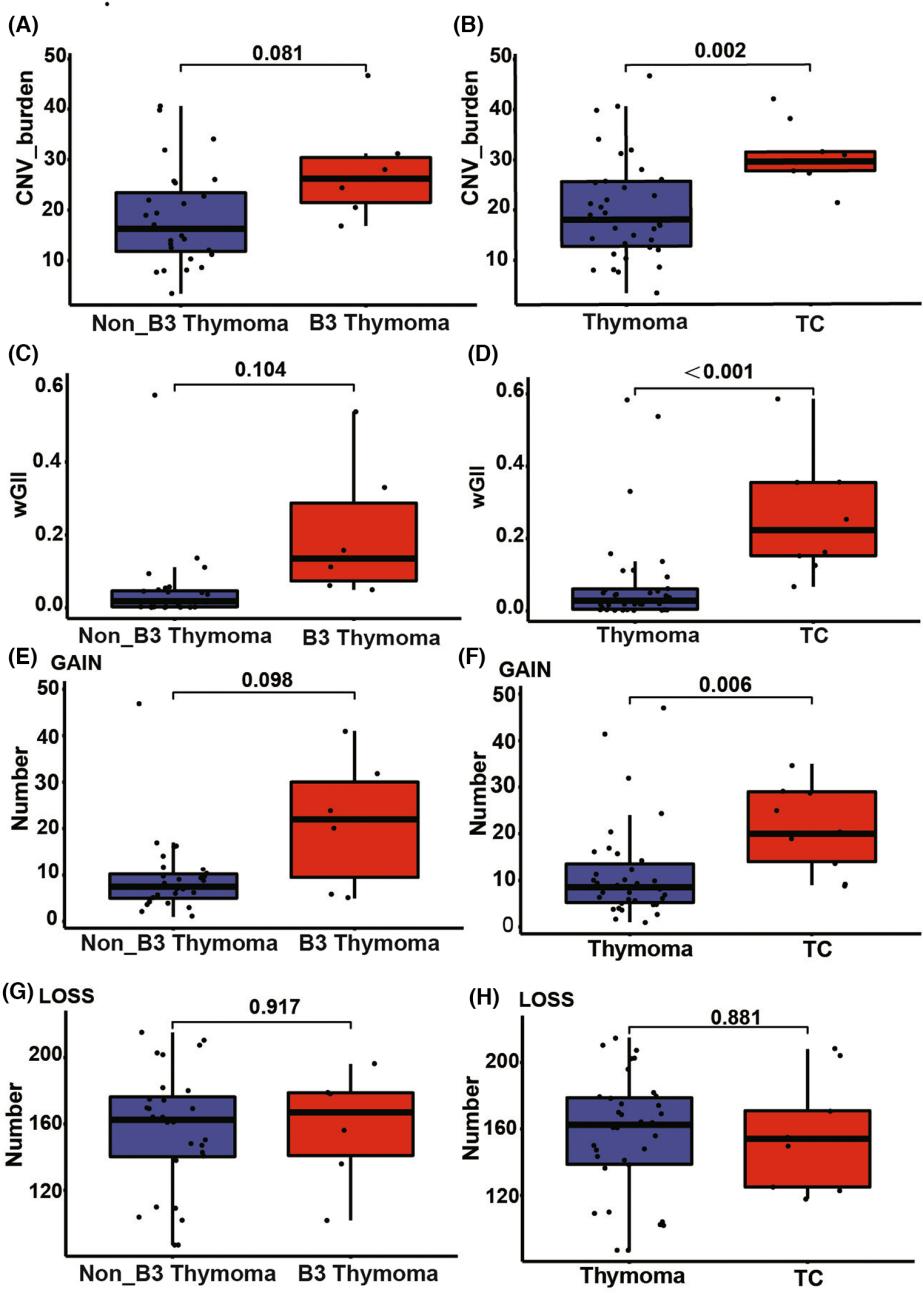
Comparison of copy number variations (CNV)‐related indicators among thymic epithelial tumors with distinct subtypes. Comparisons of (A) CNV burden, (C) wGII, (E) amplified segments, and (G) deleted segments between B3 thymoma and non‐B3 thymomas. Comparisons of (B) CNV burden, (D) wGII, (F) amplified segments, and (H) deleted segments between thymomas and thymic carcinoma. Gain, amplified segments; Loss, deleted segments.

The CNV burden values of different subtypes were shown in Figure S1A and Table 2). The CNV burden of TCs was significantly higher than that of thymomas (29.51 vs. 18.05, *p* = 0.002). Similarly, the CNV burden of B3 thymoma trended to be higher than non‐B3 thymomas (26.22 vs. 16.28, *p* = 0.081) (Figure 5A,B). As to the weighted genome instability index (wGII) (Figure S1C and Table 2), TCs had a higher value than thymomas (0.22 vs. 0.03, *p* <0.001), and type B3 thymoma tended to have a higher value than non‐type B3 thymomas (0.14 vs. 0.02, *p* = 0.104) (Figure 5C,D). However, there was no significant difference between B3 thymoma and TCs regarding either the CNV burden or wGII (Figure S1B–D).

Furthermore, the numbers of amplified and deleted segments of different subtypes were investigated separately, which were displayed in Figure S1E,G, and Table 2. On the one hand, the median number of amplified segments was remarkably higher in TCs than total thymomas (20.00 vs. 8.50, *p* = 0.006), and trended to be higher in B3 thymoma than non‐B3 thymomas (22.00 vs. 7.50, *p* = 0.098) (Figure 5E,F). On the other hand, subgroup analyses revealed no significant difference in the median number of deleted segments either between TCs and thymomas or between B3 thymoma and non‐B3 thymomas (Figure 5G,H). There was also no significant difference between B3 thymoma and TCs regarding the number of amplified and deleted segments (Figure S1F–H).

### 3.4 Comparison of signaling pathways enriched in TETs by distinct subtypes

**FIGURE 6:**
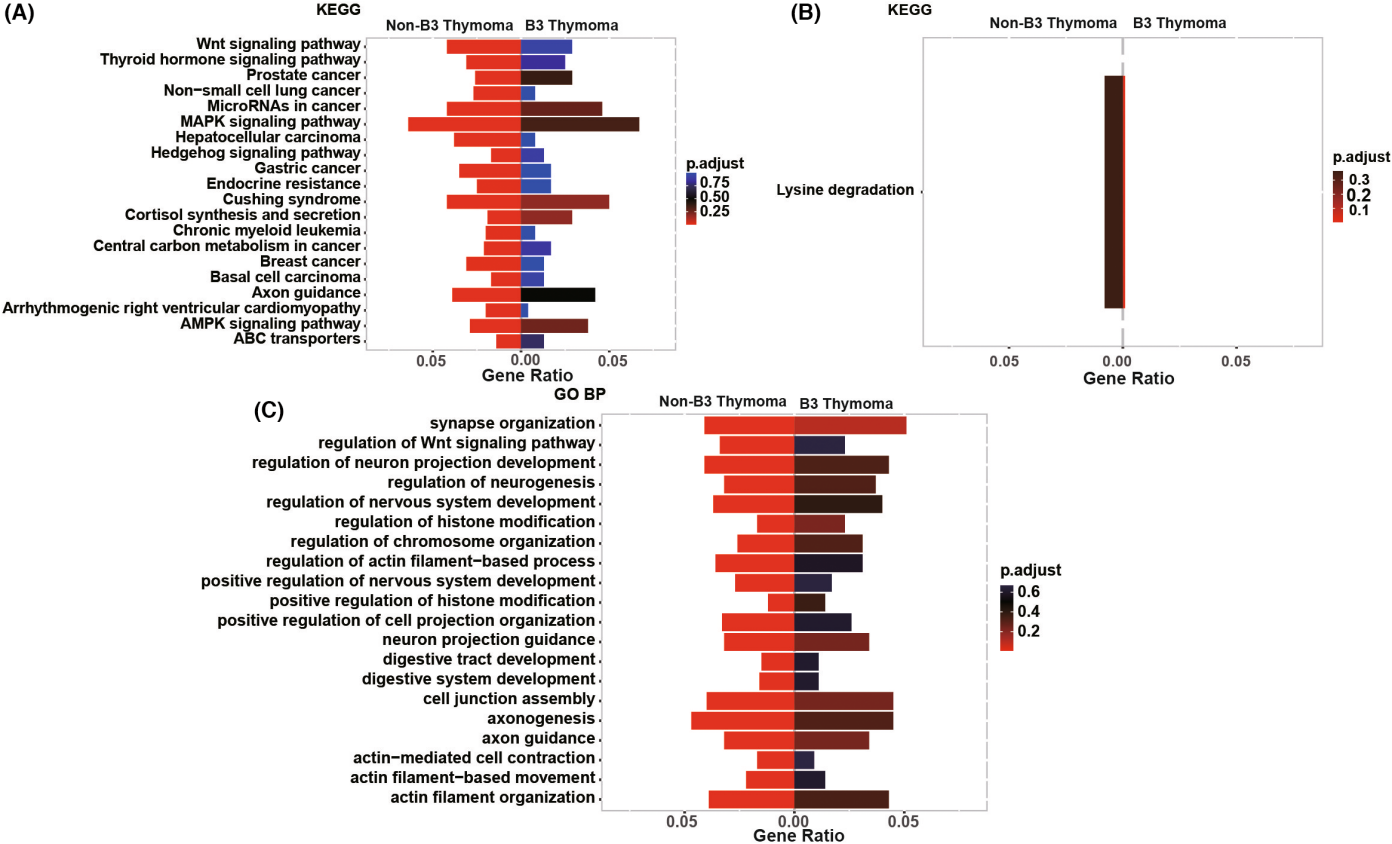
Comparison of enriched pathways between B3 thymoma and non‐B3 thymomas. (A) The pathways exclusively enriched in non‐B3 thymomas by Kyoto Encyclopedia of Genes and Genomes (KEGG) analysis. (B) The pathways exclusively enriched in B3 thymoma by KEGG analysis. (C) The pathways exclusively enriched in non‐B3 thymomas by GO biological process analysis. GO BP, GO biological process.

We further explored and compared the enriched signaling pathways in subgroups of TETs using GO and KEGG clustering analysis. The significantly enriched biological processes and pathways of type B3 thymoma and non‐B3 thymomas were shown in Figure S2A–D, respectively. The specifically‐enriched signaling pathways in non‐B3 thymomas included Wnt, MAPK, Hedgehog, AMPK, and cell junction assembly signaling pathways (Figure 6A–C), while the lysine degradation pathway was exclusively enriched in B3 thymoma (Figure 6B).

**FIGURE 7:**
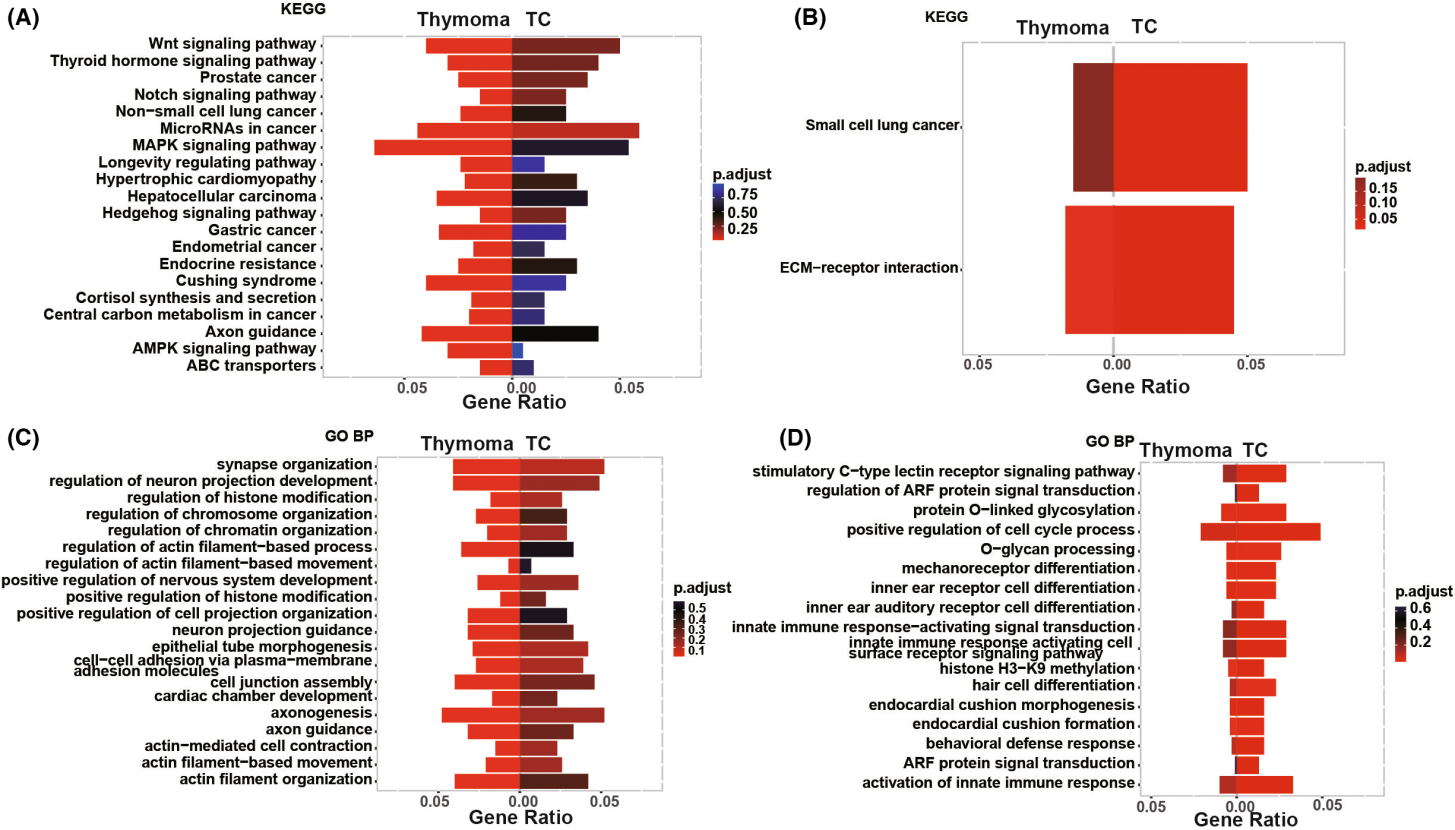
Comparison of enriched pathways between thymomas and TCs. (A) The pathways exclusively enriched in thymomas by KEGG analysis. (B) The pathways exclusively enriched in TCs by KEGG analysis. (C) The pathways exclusively enriched in thymomas by GO biological process analysis. (D) The pathways exclusively enriched in TCs by GO biological process analysis. GO BP, GO biological process.

**FIGURE 8:**
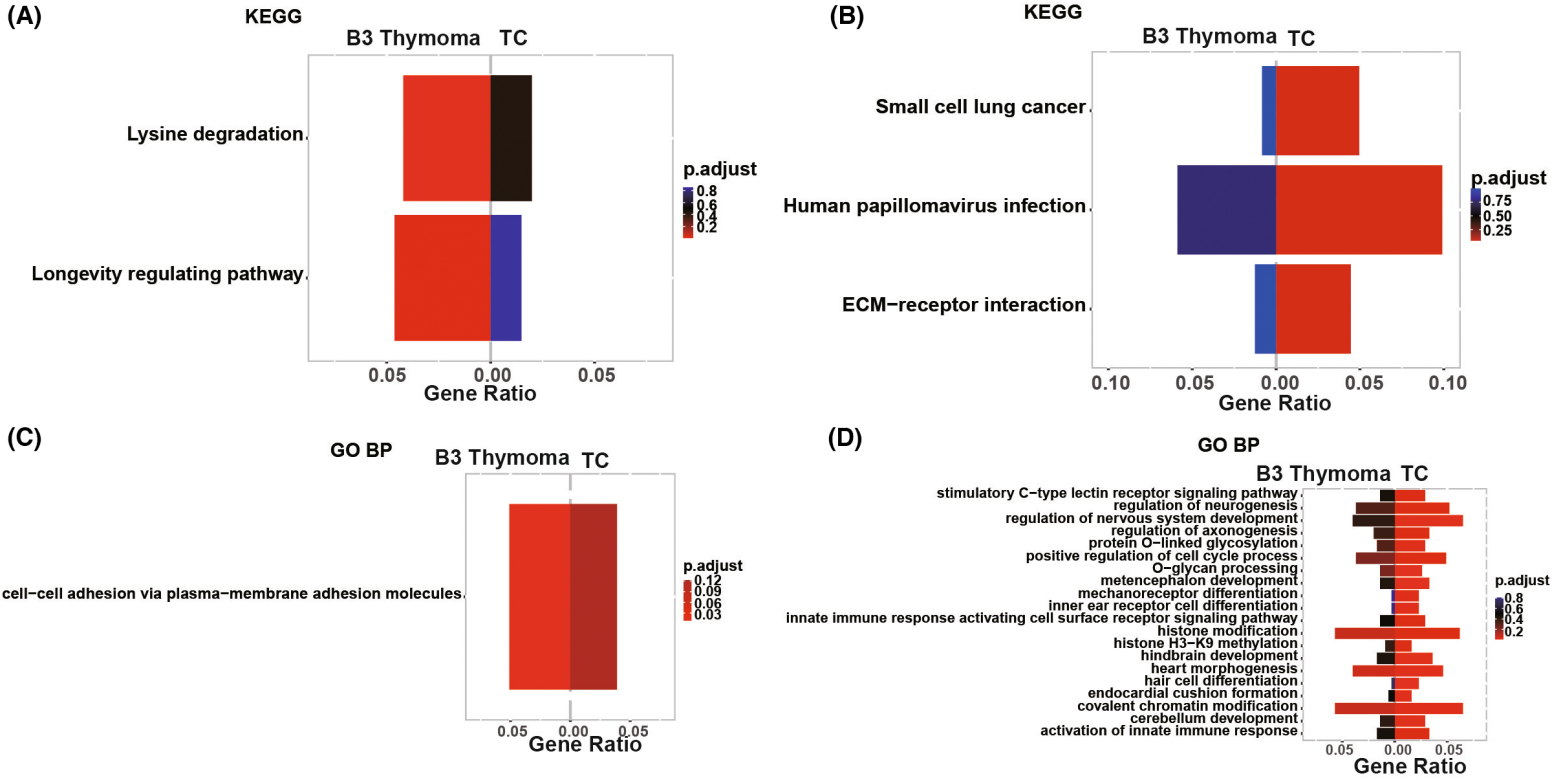
Comparison of enriched pathways between B3 thymoma and thymic carcinoma (TCs). (A) The pathways exclusively enriched in B3 thymoma by Kyoto Encyclopedia of Genes and Genomes (KEGG) analysis. (B) The pathways exclusively enriched in TCs by KEGG analysis. (C) The pathways exclusively enriched in B3 thymoma by GO biological process analysis. (D) The pathways exclusively enriched in TCs by GO biological process analysis. GO BP, GO biological process.

The significantly‐enriched biological processes and pathways of TCs and thymomas were illustrated in Figure S3A–D, respectively. The two groups shared covalent chromatin modification and histone modification pathways. Meanwhile, the unique pathways enriched in thymomas included MAPK, Wnt, AMPK, Notch, Hedgehog, and cell junction assembly signaling pathways (Figure 7A–C). In contrast, TCs were characterized by exclusive enrichments of ECM‐receptor interaction, positive regulation of the cell cycle process, and activation of innate immune response pathways (Figure 7B–D). In addition, homophilic cell adhesion via plasma membrane adhesion molecules pathway was shared by B3 thymoma and TCs, while the unique pathways enriched in B3 thymomas (Figure 8A–C) and TCs (Figure 8B–D) varied as displayed in Figure 8.

## 4 DISCUSSION

Due to the rarity and histological heterogeneity of TETs, the molecular features and underlying carcinogenic mechanisms of TETs are yet to be fully elucidated, especially for different subtypes of TETs. In this study, we analyzed and compared the mutation landscapes, TMB, CNV‐related indicators, and enriched pathways in 43 TETs with distinct histological subtypes based on the WES data.

Our study revealed several highly mutated driver genes including *PABPC1*, *ZNF208*, *PAK2*, *ZNF626*, *GTF2I*, *KMT2D*, *NOTCH1*, and *RB1*. Of note, all four patients harboring *GTF2I* mutations in the current study were diagnosed with thymoma, supporting *GTF2I* as a thymoma‐specific oncogene identified by previous studies.^7^, ^9^, ^16^ The *TP53* mutation rate was found to be 4.7% in this study, consistent with the reported frequency ranging from 3% to 14%.^5^, ^8^, ^9^, ^16^ Intriguingly, the mutation frequencies of *CDC27* and *CTNND2* trended to be higher in type B3 thymomas than in non‐B3 thymomas, implying that these cell proliferation and invasion‐associated gene^17^, ^18^ alterations may explain the more malignant features of the B3 subtype. As we know, the encoded protein by *MYO16* has been proposed to act as a serine/threonine phosphatase‐1 targeting or regulatory subunit involved in the cell cycle and cell proliferation.^19^ Notably, TCs had a distinct mutation profile with a higher mutation frequency of *MYO16* than thymomas and no *ZNF729* mutations in this study. The mutation frequency of *MYO16* also had a trend to be higher in TCs than in B3 thymoma (33% vs. 0%). Whether it may aid in distinguishing B3 thymomas from TCs or not warrants further studies with more patients to validate the specificity of *MYO16* alterations in TCs. Other recurrently mutated novel genes (e.g., ZNF729) detected in this study also deserve more in‐depth exploration of their clinical significance in TETs. If possible, it would be interesting in future studies to develop a diagnostic model based on the combination of these gene mutations.

TMB, generally defined as the number of somatic mutations per megabase of the interrogated genomic sequence, is believed to indicate immunotherapeutic response.^20^ In this study, the median TMB of TCs was only a little higher than that of thymomas (0.66 vs. 0.51) with no significant difference observed. Similarly, some other studies with relatively small sample sizes of TETs also showed no significantly higher TMB in TCs.^16^, ^21^ Large‐scale studies are warranted to confirm this finding.

A high CNV burden is associated with aggressive biological behaviors and adverse prognosis in multiple solid tumors such as high‐grade intracholecystic papillary neoplasms, pancreatic ductal adenocarcinoma, metastatic prostate cancer, and non‐small cell lung cancer.^22^, ^23^, ^24^, ^25^ In this study, CNV‐related indicators, that is, CNV burden and wGII, were higher in TCs than thymomas and trended to be higher in B3 thymoma than in non‐B3 thymomas, which suggested that high CNV burden might be involved in the aggressiveness of TETs. Interestingly, a similar trend was observed for the number of amplified segments rather than deleted segments between TCs and thymomas, B3 and non‐B3 thymomas, indicating that the differences in CNV burden were largely attributed to the number of amplified segments. Besides, CNV analyses demonstrated a similar CNV burden and wGII between B3 thymoma and TCs, suggesting their molecular overlaps to some extent and thus supporting some previous studies that classified TCs and type B3 thymoma into the same entity.^5^, ^26^ Based on this finding, we proposed that type B3 thymoma should be paid special attention to in clinical practice.

GO and KEGG clustering analysis revealed that Wnt, MAPK, Hedgehog, AMPK, and cell junction assembly signaling pathways were exclusively enriched in non‐B3 thymomas. Consistent with our finding, Yang et al. also reported that MAPK signaling was restricted to the thymomas.^16^ Considering the crucial roles of aforementioned pathways in coordinating cell growth, differentiation, migration, or metabolism,^27^, ^28^, ^29^ it seemed rational that dysregulation of these pathways may be involved in the carcinogenesis of non‐B3 thymomas. Different from non‐B3 thymomas, TCs were specifically enriched by pathways associated with ECM‐receptor interaction, positive regulation of the cell cycle process, and activation of innate immune response, which may promote uncontrollable cell proliferation and immune evasion once dysregulated.^30^ Based on the above, the discovery of these signaling pathways may eventually facilitate our understanding of various carcinogenic mechanisms underlying different subtypes of TETs, and provide the basis for potential individualized therapy.

Our study has several limitations. First, the sample size was relatively small due to the low incidence of TETs. Second, the prognostic significance of the mutational characteristics was not evaluated on account of the short follow‐up period for TETs. The patients enrolled in this study will be followed up for information on long‐term outcomes in the future.

## 5 CONCLUSIONS

Molecular profiles and enriched signaling pathways varied among distinct subtypes of TETs, which suggested diverse patterns in the pathogenesis of non‐B3 thymomas, B3 thymomas, and TCs. No difference was observed regarding the median TMB across different subtypes. Both the CNV burden and wGII were higher in TCs than thymomas, while they had no significant differences between B3 thymoma and TCs, indicating special attention should be paid to B3 thymoma in clinical practice. These findings warrant further validation in future large‐scale studies.

## AUTHOR CONTRIBUTIONS

**Jun Yang:** Conceptualization (lead); data curation (lead); investigation (lead); methodology (equal); writing – original draft (lead). **Biao Zhang:** Data curation (lead); formal analysis (lead); investigation (equal); writing – original draft (equal). **Wenyan Guan:** Data curation (lead); investigation (equal); methodology (equal). **Zhiwen Fan:** Investigation (equal); methodology (equal); software (equal). **Xiaohong Pu:** Data curation (equal); investigation (equal); methodology (equal). **Linyue Zhao:** Investigation (equal); methodology (lead). **Wen Jiang:** Investigation (equal); methodology (equal). **Weijing Cai:** Formal analysis (lead); methodology (equal). **Xueping Quan:** Formal analysis (lead); methodology (equal). **Shuying Miao:** Data curation (equal); investigation (equal); methodology (equal); software (lead). **Ling Nie:** Formal analysis (equal); investigation (equal); methodology (lead); supervision (equal). **Lu He:** Conceptualization (lead); formal analysis (equal); investigation (equal); methodology (lead); writing – review and editing (lead).

## CONFLICT OF INTEREST STATEMENT

The authors have no conflicts of interest to declare.

## ETHICS STATEMENT

All methods were performed following the Declaration of Helsinki. This study was approved by the Ethics Committee of Nanjing Drum Tower Hospital, Nanjing University Medical School (Approval No. 2021‐213‐01). Written informed consent was obtained from all study participants.

## Supporting information


Figure S1.

Figure S2.

Figure S3.

## Data Availability

All data generated or analyzed during this study are included in this published article.
