# Supplementary figures and images for: Molecular genetic characteristics of thymic epithelial tumors with distinct histological subtypes
Source: Cancer Med. 2023 Mar 14;12(9):10575–86. doi: 10.1002/cam4.5795 (PMC10225241; doi:10.1002/cam4.5795)

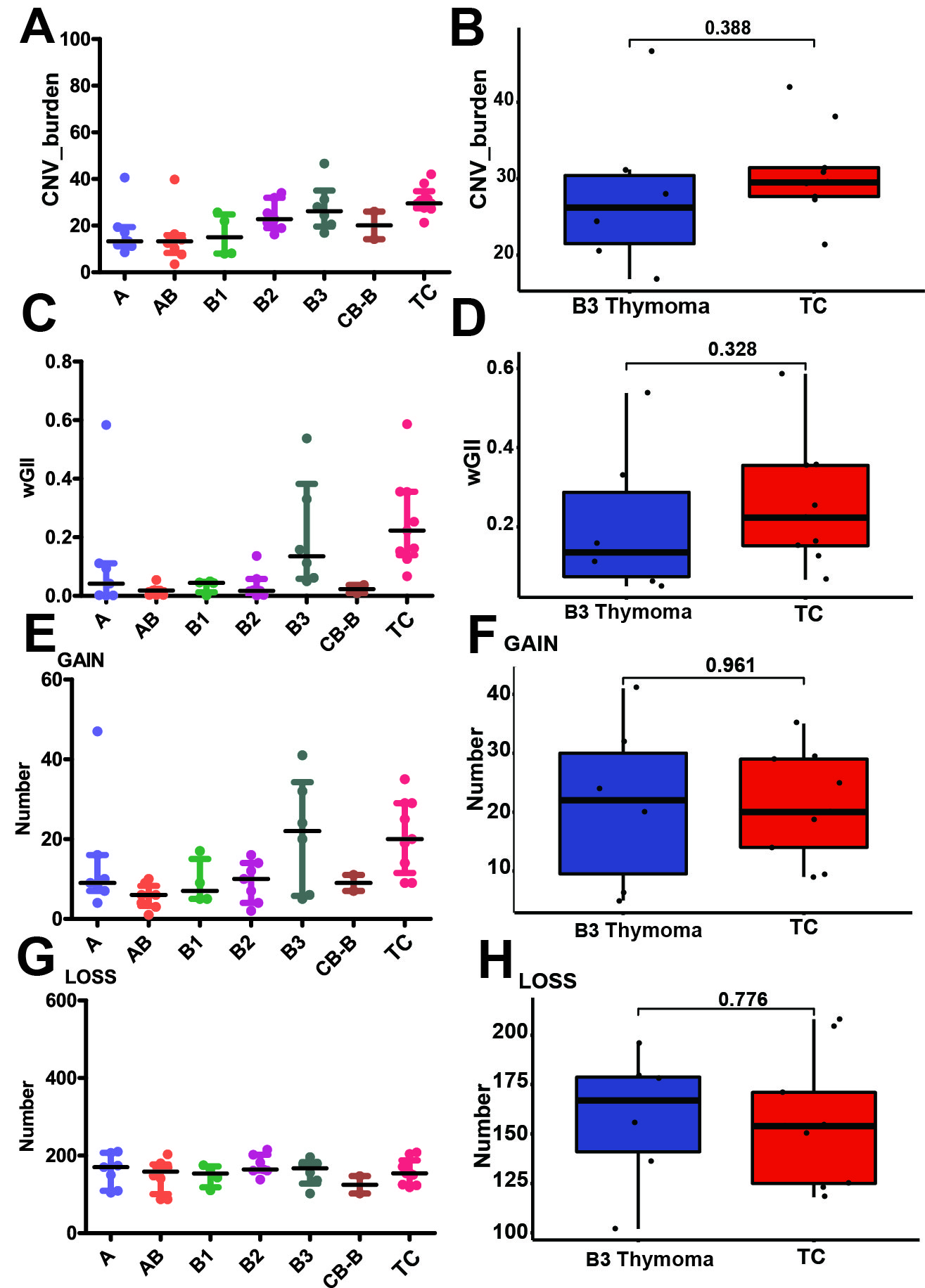

Supplement: Supplementary file 1 — Figure S1. [file CAM4-12-10575-s001.jpg]

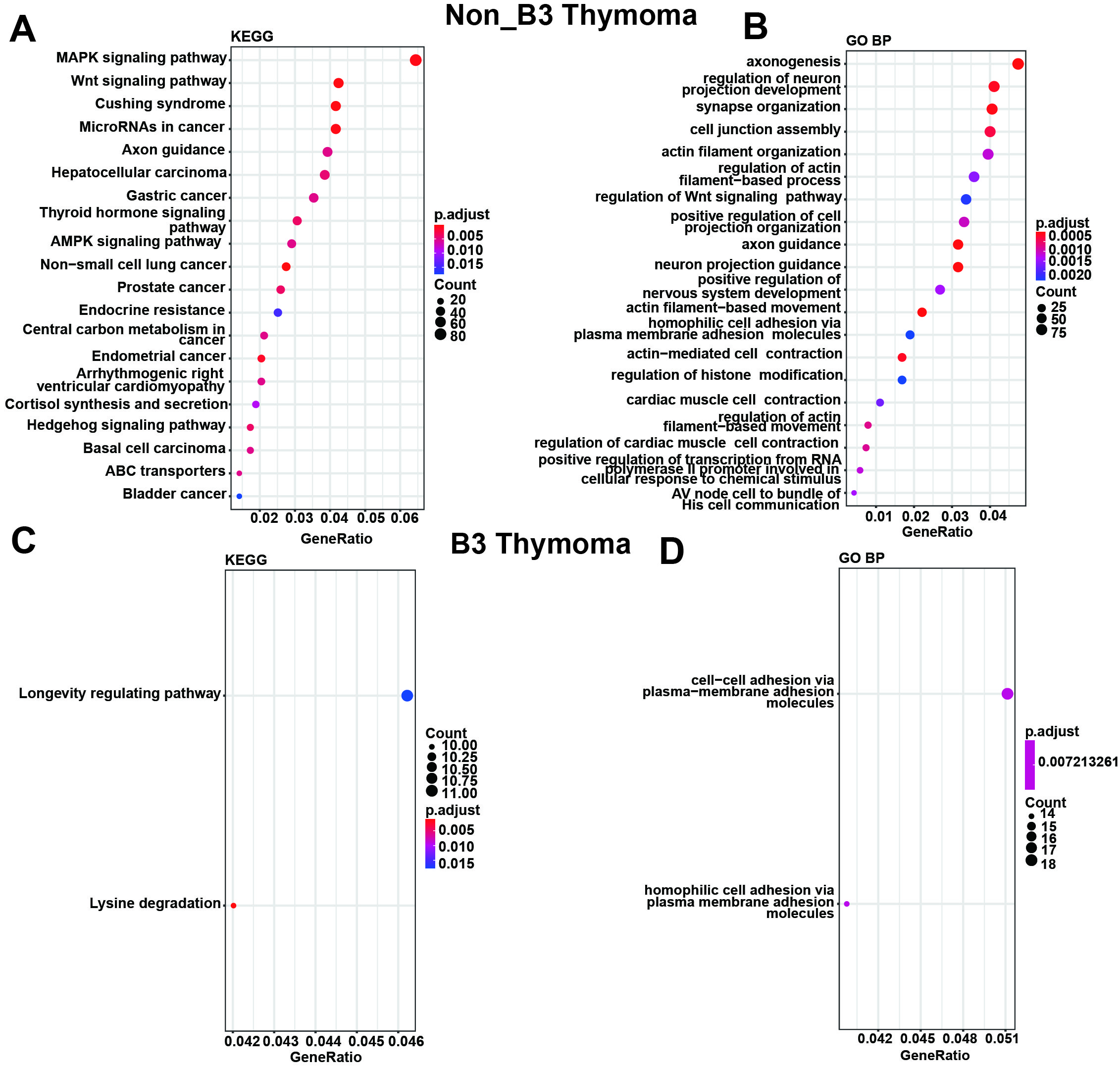

Supplement: Supplementary file 2 — Figure S2. [file CAM4-12-10575-s003.jpg]

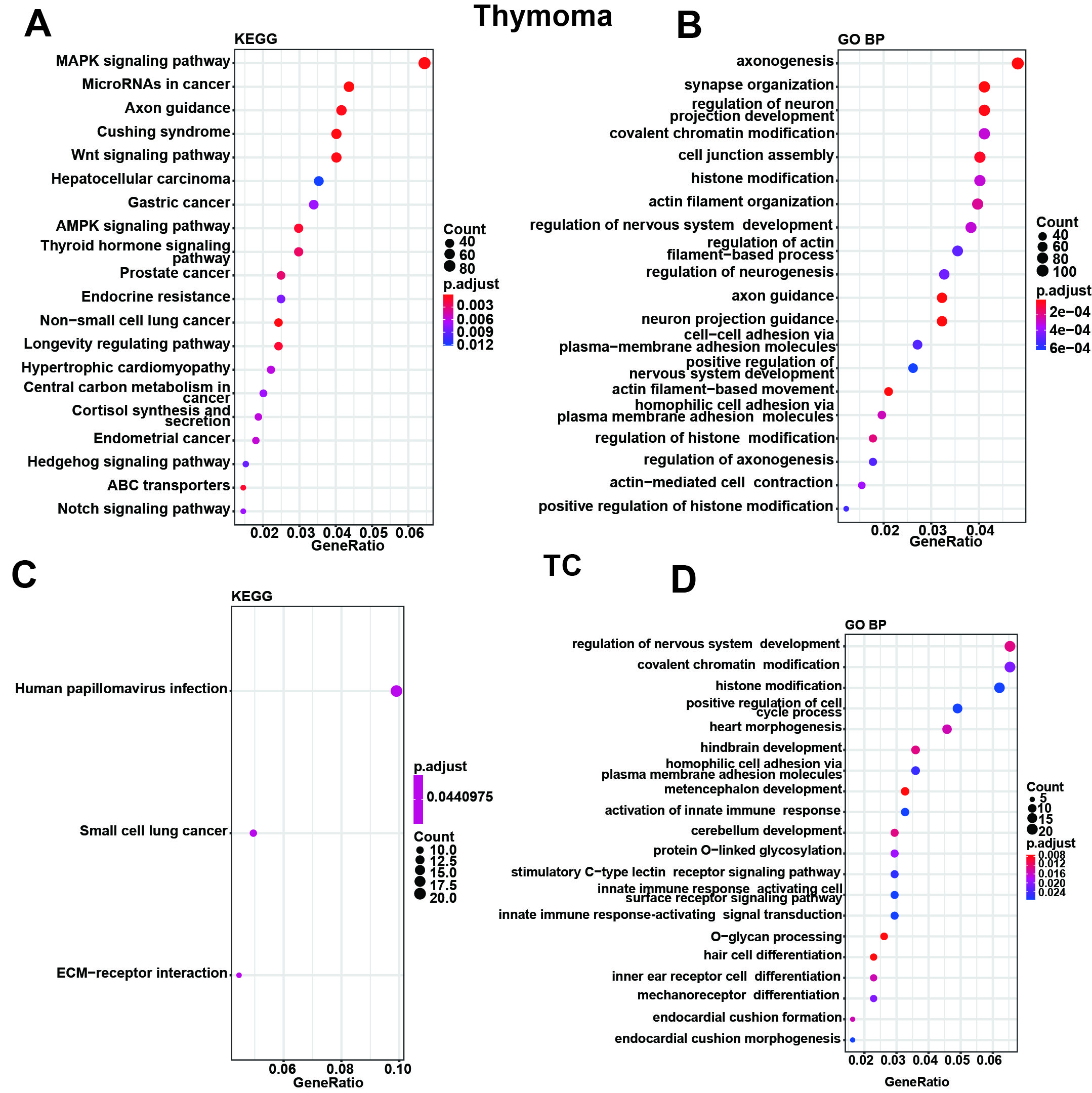

Supplement: Supplementary file 3 — Figure S3. [file CAM4-12-10575-s002.jpg]
